# Awareness of and participation in mass drug administration programs used for onchocerciasis control in the Atwima Nwabiagya North District, Ghana

**DOI:** 10.1186/s41256-023-00331-0

**Published:** 2023-11-14

**Authors:** Francis Adjei Osei, Sam Kofi Tekyi Newton, Isaac Nyanor, Eugene Osei-Yeboah, Evans Xorse Amuzu, Nicholas Karikari Mensah, Obed Ofori Nyarko, Ernest Amanor, Samuel Frimpong Odoom, Suraj Yawnumah Abubakar, Mathias Dongyele, Aliyu Mohammed, Ofeibea Asare, Stephanie Boadi, Peter Furu, Dan Wolf Meyrowitsch, Ellis Owusu-Dabo

**Affiliations:** 1 Department of Global Health, School of Public Health, Kwame Nkrumah University of Science and Technology, Kumasi, Ghana; 2 Komfo Anokye Teaching Hospital, Kumasi, Ghana; 3 Ghana Health Service, Bolgatanga, Ghana; 4 Department of Epidemiology and Biostatistics, Kwame Nkrumah University of Science and Technology, Kumasi, Ghana; 5 Career Development Centre, Kwame Nkrumah University of Science and Technology, Kumasi, Ghana; 6 Department of Public Health, Global Health Section, University of Copenhagen, Copenhagen, Denmark

**Keywords:** Mass drug administration, Onchocerciasis, Awareness, Ghana

## Abstract

**Background:**

Studies on Mass drug administration (MDA) in Ghana targeting various diseases, have mostly focused on factors that affect coverage and compliance to MDA with limited focus on evidence regarding awareness and community perception of the program. Therefore, this study sought to provide empirical evidence on the knowledge of onchocerciasis, and awareness of and participation in the MDA among community members.

**Methods:**

A community-based cross-sectional survey was conducted from August to October 2019 in communities within the Atwima Nwabiagya North District, Ghana. Data was collected from 2,008 respondents. Bivariate and multivariate logistic regression analyses were performed to measure the associations between socio-demographics, having heard of onchocerciasis and its prevention, and levels of awareness of the MDA program.

**Results:**

A total of 1268 respondents (63.2%) were aware of the MDA program. The majority ofMost respondents (74.4%) were of the view that the information given about the program was not enough and 45.4% of the respondents had no idea about the relevance of the MDA program. Respondents who had ever heard about onchocerciasis prevention and persons who had previously participated in the MDA program were more likely to be aware of the MDA program during implementation (AOR = 2.32; 95% CI 1.79–3.01 and AOR = 9.31; 95% CI 7.06–12.26, respectively).

**Conclusions:**

We observed a significant association between being aware of MDA campaigns and knowledge of onchocerciasis and its preventive methods, and participation in previous MDA campaigns. We recommend intensification and improvement of prevention campaigns regarding the onchocerciasis MDA program as key to ensuring increased MDA program participation.

## Background

Onchocerciasis, commonly known as river blindness is a vector-borne disease caused by the filarial nematode *Onchocerca volvulus* and transmitted by a black fly of the genus *Simulium*. The disease occurs mainly in the tropics with an estimated 99% of all infected persons living in sub-Saharan Africa [1]. Onchocerciasis accounts for a significant proportion of blindness and skin morbidities [2–4] Mass drug administration (MDA) of ivermectin was introduced in Ghana in 1995 as a Community Directed Treatment approach intended to eliminate onchocerciasis in Ghana and other endemic African countries [3].

Ivermectin since its introduction, more than two decades ago, has proven to be a safe and effective preventive measure against onchocerciasis [5]. Despite the substantial success gained from the introduction of MDA, onchocerciasis remains endemic in some African communities. The prevalence of onchocerciasis in sub-Saharan Africa ranges from 2.1% to 51.0% in endemic areas [6–8]. In Ghana, onchocerciasis is a considerable public health problem, with a reported prevalence of microfilaria positivity range of 2.4% to 13.2% [9] A study by Lamberton et al. [6] recorded infection with *Onchocerca volvulus* in 2.3% of black flies captured in endemic communities surveyed in Ghana. Atwima Nwabiagya District in the Ashanti Region is one of the onchocerciasis endemic districts in Ghana.

MDA offers the potential to facilitate the elimination of onchocerciasis. It has been established that the continued MDA of ivermectin is efficacious in serving as a microfilaricide and an adult worm sterilizer [8]. Treatment with ivermectin kills microfilaria and subsequently inhibits the release of microfilaria by adult female worms [7]. MDA for the eradication of helminthic diseases such as onchocerciasis, schistosomiasis, and lymphatic filariasis was designed to operate as a community-driven initiative with minimal involvement by health workers [10, 11]. This community-based intervention engages Community Drug Distributors (CDDs) who are residents in the communities to promote the acceptability of MDA among community members.[12]. In 2018, Ghana recorded a high ivermectin MDA coverage of 81.0% of persons living in onchocerciasis endemic areas [13]. However, in 2017 the MDA coverage for Atwima Koforidua and Owabi communities were below the Ghana target coverage of 80% (46.9% and 71.5% respectively) [13].

Knowledge of MDA is a useful measure of the onchocerciasis elimination agenda. Studies have demonstrated the link between knowing about MDA and taking ivermectin [14, 15]. Ivermectin MDA is currently among the acceptable means of eliminating onchocerciasis, however realizing the community’s perceived risk of the infection affects acceptability and participation [16]. It is worth noting that the number of years of MDA implementation does not guarantee awareness among community members [17]. It is unclear whether the low coverage observed at the study sites was influenced by inadequate awareness and perception about the MDA program.

Previous studies on MDA have mostly focused on factors that affect compliance with the MDA program, but little information exists on the level of awareness of the MDA program among community members, which could influence the level of acceptability and compliance. The main objective of the present study, therefore, was to examine the awareness and perception of mass drug distribution among community members in Atwima Nwabiagya District. More specifically, the survey assessed the associations between selected demographic, socio-economic predictors, onchocerciasis knowledge-related predictors, and awareness of the MDA program.

## Methods

### Study design and setting

A community-based, cross-sectional survey was conducted from August 2019 to October 2019 in two peri-urban communities and two rural communities in the Atwima Nwabiagya District of the Ashanti Region, Ghana. Household data was collected using a questionnaire and this was done immediately after the 2019 round of MDA.

Atwima Nwabiagya North District, one of the 43 districts of the Ashanti Region, was purposely selected as a study area that did not meet the expected geographical coverage of the MDA target of 80% during the 2016 and 2017 treatment campaigns (74.2% and 74.6% achieved respectively) [13]. Two rural communities in this district (Owabi and Daabaa) and two peri-urban communities (Koforidua and Ntensere) were selected for the study. The choice of rural and urban was to ensure a fair representation of the different socio-economic and cultural backgrounds. All four communities were located along the Owabi dam, and they were purposely selected for the study due to reports of high intensity of transmission of *O. volvulus* in these communities despite over two decades of participating in the MDA campaigns [13]

The Owabi dam is located 10 km northwest of Kumasi, the capital town of the Ashanti Region. The Owabi catchment area covers an area of 69 km^2^ [18] and has a population of 63,154 individuals [19]. The Owabi River flows through agricultural land close to the surrounding villages and it serves as the main source of water for farming purposes.

It is estimated that 67% of the working population in the district is engaged in agriculture. The main cash crops cultivated are cocoa, citrus, and oil palm whilst maize, cassava, plantain, cocoyam, and rice are the major food crops produced in the district. The other major economic activities are manufacturing, services, and commerce [19]. A report from the district assembly suggests high poverty levels, especially among women due to low literacy rates, and income disparities between rural and peri-urban settings [20].

### Study population and sampling

The study included all persons aged 18 years and above who had lived in the study communities for more than three months before data collection. This target group was included in the study because minors (aged 17 years or below) although eligible have relatively low knowledge of the MDA exercise. Also, in Ghana, persons 18 years and above are of adult age and can accordingly give informed consent, which was a mandatory requirement for this study. The study considered persons who had lived in the community for more than three months because the most recent MDA had been conducted less than three months before this study. It was expected that the respondents would have the knowledge and the opportunity to participate in the just-ended MDA campaign, hence the decision to include them.

The awareness rate of the MDA program was projected at 41.0% [21], and a ratio of 1:1 respondent was set among being aware of the MDA program or not, and using a power of 80%, a 95% confidence interval and 10% non-response rate. A minimum sample size of 1700 was estimated. 2008 study subjects were recruited. The households in the communities (Owabi and Daabaa) were randomly selected using a simple random sampling technique. In the peri-urban communities (Koforidua and Ntensere), each community was demarcated into six clusters. Two clusters from each community were selected using a simple random technique, and then a systematic sampling technique was used to select households for the survey. Thus, in each household, one person (either the household head or a household member aged eighteen or above) was selected and interviewed.

### Data collection

Information collected from respondents included socio-demographic and economic factors, awareness of the MDA program, sources of information on MDA, preferred channels of information on MDA, and their perception of the benefits of the drugs dispensed with specific reference to MDA against onchocerciasis.

Data were collected using a pre-coded, structured interviewer-administered questionnaire with twenty-eight questions. An electronic database was designed using the Research Electronic Data Capture (REDCap®) system hosted at the Kwame Nkrumah University of Science and Technology [22].

Ten data collectors were trained to administer the questionnaires and conduct the interviews. Training of the data collectors included a review of the questionnaires and interview guides, field-based training, and a pilot run of the questionnaires and interview guides in an adjacent community.

### Exposure and outcome variables

The outcome variable was awareness of the MDA campaign, defined as whether the community member expressed having heard about the onchocerciasis MDA activity that was conducted in the study community during the 2019 round of MDA.

Exposure variables were the determinants of MDA awareness in a low-resource setting like Ghana. These were selected a priori based on previous studies [14–16]

The respondents were categorized into age groups of 18–29 years, 30–39 years, 40–49 years, 50–59 years, and 60 years and above. Marital status was categorized into single, separated, and living with a partner. Employment status was categorized into unemployed, skilled, and unskilled, and education into no formal education, basic (from primary school to junior high school), secondary (respondents who had completed senior high school), and tertiary (participants who had attained a post-secondary education).

The duration of living in the community was categorized into < 10 years and ≥ 10 years. A wealth index was constructed for the socio-economic status index of the study respondents from household asset data using principal components analysis and categorized as low and high [23].

The socio-economic variables used to determine the socio-economic status of the respondents were: electricity in the household, a bank account, radio, television, covered by health insurance, mobile phone, owning any livestock, other farm animals or poultry, refrigerator, own any agricultural land, own car/truck, sewing machine, computer, motorcycle, cement/ceramic/marble/porcelain tiles/terrazzo as flooring materials, electricity/LPG/natural gas/biogas as fuel for cooking, pipe/borehole/protected well/bottle/sachet water, flush/pour flush toilet facility, type of roofing (metal/wood/ceramic/brick tiles/slate/asbestos/sheets). These materials were coded as one if an individual has the household asset or otherwise zero.

Each asset was assigned a weight (factor score) generated through principal components analysis, [24], and the resulting asset scores were standardized to a normal distribution with a mean of zero and a standard deviation of one. Each household was assigned a score for each asset, and the scores were summed for each household; individuals were ranked according to the total score of the household in which they resided. The sample was divided into two quintiles (low and high).

Other exposure variables measured were Source of information on M DA, ever heard of onchocerciasis disease, perception of the benefits (knowing that the MDA drug prevents a disease), knowledge of onchocerciasis prevention, and participation in the 2019 MDA. The knowledge of onchocerciasis was assessed by asking sets of questions depicting the knowledge of onchocerciasis. The questions were about the mode of transmission of onchocerciasis, the body part(s) affected by onchocerciasis, signs, and symptoms of onchocerciasis, vulnerable groups most affected by onchocerciasis, and the common complication(s). These responses were scored and categorized as knowing onchocerciasis or not. Questions on the adequacy of information given to respondents were determined by finding out the extent to which the information covered when (the period) of the MDA exercise, the target disease/condition, the target population, and the benefit of the MDA exercise. The results were scored and categorized into having received enough information on the MDA (Yes) or not (No).

### Data analysis

The data were analysed using Stata 16.0 statistical software (StataCorp. 4905 Lakeway Drive Station, Texas 77,845, USA).

Descriptive data were presented in frequency tables. Bivariate and multivariate analyses were used to measure the associations between exposure variables and the outcome variable. Results were reported as odds ratios (ORs) and 95% confidence intervals around the respective ORs. Multivariate logistic regression models were fitted using a backward stepwise approach to adjust for the effect of confounding factors.

## Results

A description of the respondents is given in Table 1. Half of the 2,008 respondents (50.3%) were recruited from Koforidua. 1,370 (68.2%) were females. Almost half (48.5%) of the respondents were in the age group 18–29 years, whereas 9.4% were 60 years and above. The largest proportion of respondents was engaged in trading (28.4%), 21.0% were unemployed and 15.8% were artisans. Nearly equal proportions of the respondents were married (41.4%) and single (41.8%). Most respondents had completed basic education (59.6%), whereas 13.5% reported that they had no formal education. More than half of the respondents (58.7%) had stayed in the community for at least ten years. Half of the respondents were classified as being in the low quintiles (50.1%) according to the household wealth index.

**Table 1:** Demographic characteristics of respondents

Variables	Frequencies (N = 2008)	Percentage (%)
Age (years)		
18–29	974	48.5
30–39	415	20.7
40–49	279	13.9
50–59	151	7.5
60 and above	189	9.4
Sex		
Female	1,370	68.2
Male	638	31.8
Occupation		
Unemployed	422	21.0
Fisherman/Farming	201	10.0
Trading	570	28.4
Artisan	318	15.8
Civil servant	90	4.5
Student/Apprentice	311	15.5
Other	96	4.8
Marital status		
Single	839	41.8
Cohabiting	131	6.5
Married	832	41.4
Divorced	98	4.9
Widowed	108	5.4
Educational status		
No formal education	270	13.5
Basic	1,196	59.6
Secondary	448	22.3
Tertiary	94	4.7
Community		
Daabaa	418	20.7
Koforidua	1,018	50.3
Ntensere	458	22.6
Owabi	129	6.4
Duration stayed in the community		
< 10 years	829	41.3
> = 10 years	1179	58.7
Household Wealth Index		
Low quantile	1005	50.1
High quintile	1003	49.9

Table 2 describes the respondents’ perceptions of the MDA program. More than half of the respondents (63.2%) were aware of the MDA program. Among the main sources of information on the MDA program, were radio (24.3%) and the Community Based Surveillance (CBS) volunteers (22.3%). Most of the respondents (74.4%) reported that the information given on the MDA was not enough. More than half of the respondents (53.6%) shared the view that the MDA program was helpful in the communities whereas less than half of the respondents (45.4%) indicated that they had no idea about the relevance or otherwise of the MDA program.

**Table 2:** Descriptive assessment of the perception of MDA

Variables	Frequencies N = 2008	Percentage (%)
Awareness of the MDA program		
No	740	36.8
Yes	1268	63.2
Source of information on the 2019 MDA*		
Radio/ Information Centre	488	24.3
Van announcement	75	3.7
Health workers	258	12.9
CBS volunteers	447	22.3
Flyers/Posters	13	0.7
Family/Friends	163	8.1
TV	19	1.0
School	137	6.8
Religious institution	14	0.7
Other	581	28.9
Was enough information given?		
No	1494	74.4
Yes	514	25.6
Preferred means of information*		
TV	675	33.6
Radio	1029	51.3
Health workers	561	27.9
CBS Volunteers	428	21.3
Van announcement	374	18.6
Community Information Center	1406	70.0
School	225	11.2
Religious institutions	240	12.0
Family/Friends	88	4.4
Other	91	4.5
Is the MDA helpful (ability to protect community members from getting sick of onchocerciasis) in the communities		
Not helpful	21	1.1
Partially helpful	144	7.4
Very helpful	905	46.2
Do not know	890	45.4

*More than one answer is allowed

Factors associated with whether a respondent is likely to be aware of the MDA program are presented in Table 3. Awareness of MDA program differed significantly in terms of sex (*p* = 0.002), education (*p* = 0.003), occupation (*p* =  < 0.001), duration of stay at the community (*p* = 0.004), knowledge of onchocerciasis prevention (*p* =  < 0.001), perceived benefit of MDA program (*p* =  < 0.001) and participation in previous MDA (*p* =  < 0.001).

**Table 3:** Factors associated with awareness of the MDA program

Variable	Not aware, n (%)	Aware, n (%)	Total, n	*p*-value
Age (years)				
18–29	365 (37.5)	608 (62.5)	973	0.464
30–39	150 (36.1)	265 (63.9)	415	
40–49	108 (38.7)	171 (61.3)	279	
50–59	58 (38.4)	93 (61.6)	151	
60 and above	59 (31.1)	131 (68.9)	190	
Sex				
Female	474 (34.6)	896 (65.4)	1370	0.002
Male	266 (41.7)	372 (58.3)	638	
Household Wealth Index				
Low quintile	362 (36.0)	643 (64.0)	1005	0.439
High quintile	378 (37.7)	625 (62.3)	1003	
Education				
No formal education	80 (29.6)	190 (70.4)	270	0.003
Basic	433 (36.2)	763 (63.8)	1196	
Secondary and above	227 (41.9)	315 (58.1)	542	
Occupation				
Unemployed	247 (33.7)	486 (66.3)	733	< 0.001
Skilled Labour	198 (48.5)	210 (51.5)	408	
Unskilled Labour	295 (34.0)	572 (66.0)	867	
Duration of stay at the community				
< 10 years	336 (40.5)	493 (59.5)	829	0.004
> = 10 years	404 (34.3)	775 (65.7)	1179	
Ever heard of onchocerciasis				
Yes	368 (27.54)	968 (72.46)	1336	< 0.001
No	372 (55.36)	300 (44.64)	672	
Can onchocerciasis be prevented?				
Yes	470 (32.84)	961 (67.16)	1431	< 0.001
No	235 (47.19)	263 (52.81)	498	
MDA drug beneficial				
Yes	469 (30.43)	1072 (69.57)	1541	< 0.001
No	147 (52.88)	131 (47.12)	278	
Participation in previous MDA				
Yes	97 (10.43)	833 (89.57)	930	< 0.001
No	643 (59.65)	435 (40.35)	1078	
Residential status				
Rural	103 (13.92)	441 (34.78)	544	< 0.001
Peri-Urban	637 (86.08)	827 (65.22)	1464	

The results of bivariate and multivariate analyses regarding predictors for awareness of the MDA program are presented in Table 4. The odds ratio indicates the likelihood of being aware of the MDA program. Respondents who had stayed in the community for 10 years and more (AOR = 0.67; 95% CI 0.52–0.86) had a reduced odds of being aware of the MDA program. Persons who had ever heard of onchocerciasis were more likely to be aware of the MDA program (AOR = 2.32, 95% CI 1.79–3.01). In addition, respondents who knew about onchocerciasis prevention were more likely to be aware of the MDA program (AOR = 1.44; 95% CI 1.08–1.93). Having participated in a previous MDA campaign significantly increased the likelihood of being aware of the MDA program (AOR = 9.31; 95% CI 7.06–12.26).

**Table 4:** Association between awareness of the MDA program, socio-demographics characteristics, and knowledge of onchocerciasis

Variable	Number of respondents who were aware (% of the total in each group)	OR (95% CI)	*p*-value	AOR (95% CI)	*p*-value
Age (years)					
18–29	608 (48.0)	Ref		Ref	
30–39	265 (20.9)	1.06 (0.84–1.35)	0.629	0.96 (0.67–1.34)	0.756
40–49	171 (13.5)	0.95 (0.72–1.25)	0.716	0.76 (0.50–1.17)	0.215
50–59	93 (7.3)	0.96 (0.68–1.37)	0.832	0.79 (0.47–1.33)	0.382
60 and above	131 (10.3)	1.33 (0.95–1.86)	0.091	0.81 (0.49–1.33)	0.408
Sex					
Female	896 (70.7)	Ref		Ref	
Male	372 (29.3)	0.74 (0.61–0.89)	0.002	0.73 (0.56–0.96)	0.026
Marital status					
Single/Separated	162 (40.8)	Ref		Ref	
Living with a spouse	235 (59.2)	0.99 (0.83–1.19)	0.945	1.12 (0.85–1.48)	0.421
Occupation					
Unemployed	103 (25.9)	Ref		Ref	
Skilled	77 (19.4)	0.54 (0.42–0.69)	< 0.001	0.84 (0.59–1.18)	0.307
Unskilled labour	217 (54.7)	0.99 (0.80–1.21)	0.890	0.99 (0.73–1.35)	0.953
Educational status					
No Formal Education	61 (15.4)	Ref		Ref	
Basic	215 (54.2)	0.74 (0.56–0.99)	0.040	0.81 (0.54–1.21)	0.301
SHS and above	121 (30.5)	0.58 (0.43–0.79)	0.001	0.67 (0.43–1.06)	0.088
Household Wealth Index					
Low quintile	643 (50.7)	Ref		Ref	
High quintile	625 (49.3)	1.07 (0.89–1.28)	0.439	0.89 (0.69–1.14)	0.366
Duration of stay in the community					
< 10 years	493 (38.9)	Ref		Ref	
> = 10 years	775 (61.1)	0.76 (0.64–0.92)	0.004	0.67 (0.52–0.86)	0.002*
Ever heard of onchocerciasis					
No	300 (23.7)	Ref		Ref	
Yes	968 (76.3)	3.26 (2.69–3.96)	< 0.001	2.32 (1.79–3.01)	< 0.001*
Knowledge of onchocerciasis prevention					
No	263 (21.5)	Ref		Ref	
Yes	961 (78.5)	1.83 (1.48–2.25)	< 0.001	1.44 (1.08–1.93)	0.013*
MDA drug beneficial					
No	131 (10.9)	Ref		Ref	
Yes	1,072 (89.1)	2.56 (1.98–3.32)	< 0.001	1.34 (0.96–1.86)	0.085
Participation in previous MDA					
No	435 (34.3)	Ref		Ref	
Yes	833 (65.7)	12.69 (9.95–16.18)	< 0.001	9.31 (7.06–12.26)	< 0.001*
Residential status					
Rural	441 (34.78)	Ref			
Peri-urban	827 (65.22)	0.30 (0.24–0.38)	< 0.001	0.45 (0.33–0.62)	< 0.001*

** p<0.001. *p<0.05

Ref: Reference point. SHS: Senior High School. MDA: Mass Drug Administration. cOR: Crude Odds Ratio. AOR: Adjusted Odds Ratio. CI: Confidence Interval

## Discussion

In the study areas, the rate of awareness regarding the current onchocerciasis MDA program was 63.2%. This finding is significantly higher as compared with a study conducted on lymphatic filariasis by Roy et al. [21] which found an MDA awareness rate of 41.0% (awareness defined the same way as in this present study), assuming that there is a negligible difference between the control approach to the two diseases.

We found that community information centers were the main source of MDA information for the respondents. Information centers in recent times have been a useful means of communication, particularly in resource-limited areas. A recent study outlined the role of community information centers to include the dissemination of information to rural areas on health, agriculture, local government, and education. [25] Despite the recognizable relevance of community communication centers, reports suggest that it is often not integrated into community mobilization and citizen engagement strategies from the onset [26]. It is worth stressing the need for stakeholders to incorporate mass communication using appropriate mediums such as community information centers to heighten awareness.

Community health workers (CHWs) and CBS volunteers form a significant proportion of the source of information on MDA. This group plays an advocacy and educational role in the MDA campaign. A study by Inobaya et al. [25] examined the critical role played by health workers during MDA campaigns. They found that health workers had poor awareness of the signs and symptoms, prevention, and the treatment options available for the NTD studied (schistosomiasis). To achieve an effective MDA campaign, CWHs (including community volunteers) must be given the necessary training and motivation to enable them to play a role in community education, mobilization, and improving community participation in MDA campaigns.

In our study, about one percent of respondents indicated that the drug was not helpful, whereas 45% reported that they did not know whether the MDA drug was helpful or not. These findings show the extent to which community members living in endemic areas had an inadequate understanding of the program after decades of implementation in their communities. This could lead to negative perceptions about the MDA program. Several studies reviewed [27–29], showed that the fear of the side effect of the drug contributes to non-acceptability and non-compliance with the MDA program. This leads to a solidified negative community knowledge and perception about the harm of the MDA drug leading to poor intervention outcomes. The implementers are required to understand this perception to address them during the education before the drug distribution [27–29].

A global survey conducted by Keenan et al. [30] among NTD experts to elicit their opinions on the relevance of MDA observed a high understanding of the programmatic goals of five NTDs, including onchocerciasis. It is however worrying that this understanding does not trickle down to the community members living in endemic areas who are the direct beneficiaries of these interventions.

In seeking to improve awareness creation for MDA programs, a systematic review conducted by Silumbwe et al. [31] reported that awareness creation is best done through innovative community health education programs, creating partnerships and collaborations, and integration with existing programs to facilitate MDA campaigns. They also recommended motivating community drug distributors (CDDs) through incentives and training to enable them to play an effective role in the advocacy of MDA-related activities. It is worth noting some of the barriers hindering the successful implementation of MDA programs to address them in future planning. These included a lack of geographical demarcations, delayed drug deliveries, inappropriate drug delivery strategies, and a limited number of drug distributors [31]. Furthermore, a recent study conducted in a rural setting in the Northern region of Ghana recommended the adoption of strong stakeholder engagement coupled with an evidence-based context-specific multi-channel community education approach with key educational messages on the knowledge of targeted Neglected Tropical Disease (NTD) and the benefits of MDA programs [32].

Efforts such as a biannual MDA campaign in hyperendemic communities [33], increasing the time allotted for the MDA campaign, and incentivizing the health workers and the CDDs undertaking this campaign should be done. Also, it is recommended that the CDDs be trained appropriately to understand the MDA drug to enable them to inform the beneficiaries during the MDA campaign. Finally, intensive education should be done before the commencement of the MDA campaign [32], as well as regular monitoring and evaluation of MDA campaigns [34].

This study has some limitations. First, it cannot be generalized to the entire onchocerciasis endemic communities due to cultural and geographical differences. Second, recall bias was a possibility since respondents were required to recall information about previously completed MDA campaigns. To minimize the recall bias, the data were collected immediately after the MDA campaign. Third, by excluding persons aged seventeen and below from the study, the views of adolescents on MDA are not represented in this study. Last, being a cross-sectional study, it has a limited ability to establish causality. Despite these limitations, the study makes a valuable contribution to the literature on implementation issues in MDA campaigns in low-resource settings.

## Conclusions

This study highlights the need for intensive health education in efforts to improve awareness of MDA. We observed a significant association between being aware of MDA campaigns and a long stay in the community, knowledge of onchocerciasis and its preventive methods, and participation in previous MDA campaigns. As we countdown toward the elimination of onchocerciasis by the year 2025 and meet the SDG target 3.3 by the year 2030, there is an urgent need for more effective MDA implementation strategies that are adapted to local needs. We recommend the use of local community information centers, and local radio stations for education during MDA campaigns. We also recommend the active involvement of key stakeholders in the community such as the educational system (schools), community leaders, and governmental and non-governmental organizations to support the campaign.

## Data Availability

The data from the study is available at the lead author’s institution and is available upon written request.

## Abbreviations

MDA: Mass drug administration
CI: Confidence interval
GDHS: Ghana demographic and health survey
OR: Odds ratio
CBS: Community-based surveillance
CHWs: Community health workers
NTD: Neglected tropical diseases
CDDs: Community drug distributors
SDG: Sustainable development goal
